# GEGA (Gallus Enriched Gene Annotation): an online tool providing genomics and functional information across 47 tissues for a chicken gene-enriched atlas gathering Ensembl and Refseq genome annotations

**DOI:** 10.1093/nargab/lqae101

**Published:** 2024-08-16

**Authors:** Fabien Degalez, Philippe Bardou, Sandrine Lagarrigue

**Affiliations:** PEGASE, INRAE, Institut Agro, 35590 Saint Gilles, France; Sigenae, GenPhySE, Université de Toulouse, INRAE, ENVT, F-31326 Castanet Tolosan, France; PEGASE, INRAE, Institut Agro, 35590 Saint Gilles, France

## Abstract

GEGA is a user-friendly tool designed to navigate through various genomic and functional information related to an enriched gene atlas in chicken that integrates the gene catalogues from the two reference databases, NCBI-RefSeq and EMBL-Ensembl/GENCODE, along with four additional rich resources such as FAANG and NONCODE. Using the latest GRCg7b genome assembly, GEGA encompasses a total of 78 323 genes, including 24 102 protein-coding genes (PCGs) and 44 428 long non-coding RNAs (lncRNAs), significantly increasing the number of genes provided by each resource independently. However, GEGA is more than just a gene database. It offers a range of features that allow us to go deeper into the functional aspects of these genes. Users can explore gene expression and co-expression profiles across 47 tissues from 36 datasets and 1400 samples, discover tissue-specific variations and their expression as a function of sex or age and extract orthologous genes or their genomic configuration relative to the closest gene. For the communities interested in a specific gene, a list of genes or a quantitative trait locus region in chicken, GEGA’s user-friendly interface facilitates efficient gene analysis, easy downloading of results and a multitude of graphical representations, from genomic information to detailed visualization of expression levels.

## Graphical Abstract

**Graphical Abstract:**
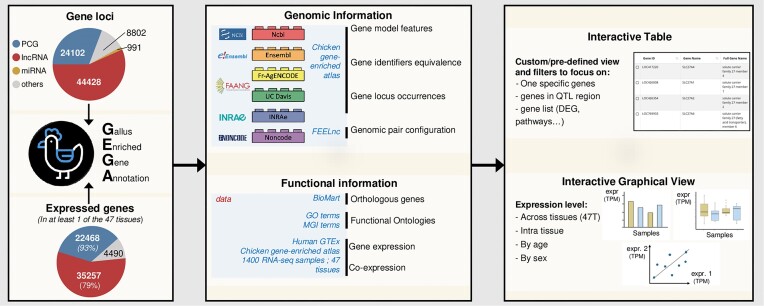

## Introduction

The chicken (*Gallus gallus*) genome is a valuable model in both fundamental and applied research (1). As a key model species, it is frequently used to investigate vertebrate development, evolution and, more generally, diseases. Additionally, the chicken is one of the livestock species which supplies the most protein-rich food worldwide through both meat and egg production. In recent years, significant progress has been made in annotating this genome with, for example, the introduction of long non-coding RNA (lncRNA) gene models in the two reference gene model databases, NCBI-RefSeq and EMBL-EBI Ensembl/GENCODE. These reputable databases provide complementary information on gene models due to different RNA-seq resources and bioinformatics pipelines used for the gene modelling (2). As previously reported (3), the overlap of protein-coding gene (PCG) models between both reference databases is ∼90%, typically with different transcript models supporting the same PCG loci. Concerning lncRNA genes, which are regulatory elements of gene expression characterized by condition-specific expression (tissue and temporal), the overlap between their loci reaches ∼25% between both annotations. By combining the two reference annotations and providing gene identifier correspondence for common gene loci, the scientific community can exploit the strengths of both databases. This integration grants access to a greater number of gene models, thereby enhancing the accuracy of analyses and facilitating the use of tools provided by each database. Despite initiatives like the MANE project for human genes (4) aiming to determine common gene models between these annotations, an annotation resulting from the union of these reference databases is not available to date. In this context, we recently released an updated gene atlas of the chicken genome based on the latest GRCg7b assembly. This atlas integrates reference annotations from both NCBI-RefSeq and EMBL-EBI Ensembl/GENCODE databases, along with gene models from additional resources such as multi-tissue projects initiated by the FAANG consortium (5–7) or lncRNA-dedicated databases such as NONCODE (8), as presented in Degalez *et al.* (3). Briefly, this gene atlas consists of 78 323 gene models including 24 102 PCGs and 44 428 lncRNAs, with a total of 63 513 (81%) genes considered as expressed [≥ 0.1 transcripts per million (TPM)] including 22 468 (93%) PCGs and 35 257 (79%) lncRNAs. Additionally, 43 252 (55%) genes are highly expressed (≥1 TPM) including 19 819 (82%) PCGs and 20 252 (46%) lncRNAs. All these genes have been functionally annotated through their expression across 47 tissues using 1400 RNA-seq samples from 36 datasets chosen to represent the diversity of chicken physiological systems. This gene atlas eases switching between EMBL-EBI Ensembl/GENCODE and NCBI-RefSeq gene identifiers, as well as between assembly versions, by providing gene identifier equivalents for the previous galgal5 and GRCg6b assemblies of the chicken. This resource is also complemented by various functional annotations, including orthologous genes in human and mouse, gene ontologies [functional Gene Ontology (GO) terms or phenotype terms] and configurations with the nearest genes.

To easily access and explore all this information (gene models, expression data and associated functional annotations), we have developed an online tool called GEGA (Gallus Enriched Gene annotation). GEGA is designed for diverse user communities, including those interested in genomic regions associated with phenotypes of interest [quantitative trait loci (QTLs)] or researchers focusing on gene expression, whether they are interested in a specific gene or a list of genes. Herein, we describe the GEGA tool and its various applications.

## Materials and methods

### Web server infrastructure

The system is built on an Apache web server, version 2.4.37, configured with OpenSSL version 1.1.1k to ensure communication security. The database server is powered by MariaDB, version 10.3.35-MariaDB, for data storage and retrieval. This software configuration was deployed on a Rocky Linux distribution.

### Back-end development

The back-end development was implemented using Node.js and with the Express.js framework. Data storage is managed with a MySQL database, with a specific instance of MariaDB version 10.3.35 selected for its compatibility and enhanced performance. These technologies facilitate efficient data manipulation and optimal handling of HTTP requests.

### Front-end development

Bootstrap version 4.6.0 was chosen as the primary CSS framework to develop the user interface, ensuring a responsive and visually appealing design for an enhanced user experience across various devices. For dynamic data manipulation and the creation of interactive tables, jQuery version 3.5.1 and DataTables version 1.10.16 were used.

### Data visualization

For data visualization, Highcharts version 10.3.3, a robust and flexible JavaScript library, was employed to create interactive and customizable charts. This integration provided a clear and intuitive visual representation of the results generated by data analysis.

The origin and processing of data used in GEGA are detailed extensively in the companion paper, Degalez *et al.* (3). Here, only a summary is provided.

### Reference assembly

The genome annotation is built on the bGalGal1.mat.broiler.GRCg7b (GCF_016699485.2) assembly of the chicken (*Gallus gallus*) genome available at https://www.ncbi.nlm.nih.gov/datasets/genome/GCF_016699485.2/.

### Data origins—individual database

The enriched genome annotation included in GEGA integrates, as detailed in Degalez *et al.* (3), gene models from six sources: the ‘NCBI-RefSeq’ (v106) (available at https://ftp.ncbi.nlm.nih.gov/genomes/all/GCF/016/699/485/GCF_016699485.2_bGalGal1.mat.broiler.GRCg7b/) and ‘EMBL-EBI Ensembl/GENCODE’ (v107) (available at https://ftp.ensembl.org/pub/release-107/gtf/gallus_gallus/) reference databases according to GRCg7b assembly and based on ∼300 (indicated at https://www.ncbi.nlm.nih.gov/genome/annotation_euk/Gallus_gallus/106/#AlignmentStats) and 60 RNA-seq (listed in data_file.txt available on https://ftp.ensembl.org/pub/release-110/mysql/gallus_gallus_gca000002315v5_rnaseq_110_6/) samples, respectively; the ‘FR-AgENCODE’ (9) and ‘FarmENCODE’ (10) FAANG pilot annotation projects (5–7) produced under GRCg6a assembly; the gene annotation from Jehl *et al.* (11) according to the galgal5 assembly and integrating gene models from different public databases complete by 364 new RNA-seq samples; and, finally, the NONCODE annotation (8) including non-coding gene models under the galgal4 assembly. Note that while the FarmENCODE project used Oxford Nanopore long-read sequencing, the five other projects mainly used short-read RNA-seq data. Gene models from assemblies older than GRCg7b were remapped using the NCBI genome remapping service with default parameters (https://www.ncbi.nlm.nih.gov/genome/tools/remap). Quality of gene models was evaluated by the degree of overlap of transcripts with FANTOM5 CAGE peaks (12) remapped from galgal5 to GRCg7b. Considering also the database popularity, gene models were added sequentially resulting in this order of aggregation: (i) RefSeq; (ii) Ensembl; (iii) FrAg; (iv) Davis; (v) Inrae; and (vi) Noncode. Gene models were sequentially added to the growing atlas if their associated transcripts did not overlap with genes already present. Overlap was defined as sharing at least one exonic base pair on the same strand, as determined using BEDtools (13). To minimize overlapping similar patterns with different biotypes, models were aggregated by biotype class.

### Data origins—expression

A set of 36 publicly available datasets, comprising a total of 1400 samples, was selected to represent a variety of 47 tissues. The list of the 47 tissues, their abbreviations and colour codes used are available on GEGA. For each tissue in each project, a median of TPM-normalized expression across samples was calculated. For tissues present in several projects, the median was calculated using the TPM medians previously calculated in each project. A gene was considered as expressed if (i) its median expression was ≥ 0.1 TPM in at least one tissue and (ii) at least 50% of samples of a tissue for a given project have a reads number ≥ 6 as well as the normalized TPM and TMM expression ≥ 0.1.

### Tissue specificity analysis

Because the tissue specificity is very sensitive to the chosen indicator, different indicators of tissue specificity are available in GEGA.

(i) The tau metrics, assessed using raw (τ) or log10 median tissue expression (τ_log10_) in TPM (14), and defined as follows:


\begin{equation*}\tau = {\mathrm{\ }}\frac{{\mathop \sum \nolimits_{t = 1}^T \left( {1 - {\mathrm{\ }}{{{\hat{x}}}_t}} \right)}}{{T - 1}};{\mathrm{\ }}{{\hat{x}}_{g,t}} = {\mathrm{\ }}\frac{{{{x}_t}}}{{\mathop {\max }\limits_{1 \le t \le T} {{x}_t}}}\end{equation*}

with ${{x}_t}$ the raw or log10 median expression of the gene of interest in the tissue $t$ and among the $T$ tissues.

A gene was considered as tissue specific for a τ or τ_log10_ ≥ 0.90; the columns ‘expr_isTS’ or ‘expr_isTSlog10’ are then equal to 1. Therefore, even if the gene is considered as tissue specific, its expression profile can vary considerably and show different breaks. A break was defined as a difference in expression by a factor of 2, i.e. fold change (FC) ≥ 2 when expression was ranked in descending order.

The number of observed breaks, associated FCs and tissues in each subgroup are indicated in the columns ‘expr_nbBreaks’, ‘expr_fcBreaks’ and ‘expr_tissueBreaks’, respectively.

(ii) The FC between the first and second most expressed tissues or (iii) the FC between the two first groups separated by a break, that was calculated in three different ways, considering: (a) the least expressed tissue in the first group and the most expressed tissue in the second one (‘expr_fcFirstBreak_lowToTop’); (b) the most expressed tissue in both groups (‘expr_fcFirstBreak_topToTop’); and (c) the median expression for both groups (‘expr_fcFirstBreak_medianGroup’).

### Classification according to the closest feature

PCG, lncRNA, microRNA (miRNA) and small RNA (including snoRNA, snRNA, sRNA, tRNA, rRNA and scaRNA) transcripts and genes were classified through their associated transcripts relative to their closest PCG and lncRNA transcript using FEELnc v.0.2.1 [default parameters, 100 kb between the transcription start site (TSS) of the transcripts of the gene of interest and the transcripts of its closest lncRNA or PCG gene model] (15). Briefly, gene pairs are split into two categories and three subcategories. Firstly, the gene of interest in the pair is considered to be ‘genic’ if it overlaps the partner gene, and ‘intergenic’ otherwise. Secondly, the gene of interest is classified according to its configuration with its partner: ‘same strand/sense’ if it is transcribed in the same orientation; ‘divergent/antisense’ if it is transcribed in head-to-head orientation; and ‘convergent/antisense’ if it is oriented in tail to tail. For each type of pairs (e.g. lncRNA:PCG denoted ‘LncPcg’ in which the lncRNA is the gene of interest), information such as the class name of the configuration according to FEELnc (e.g. lncgSSinNest, which means that the lncRNA gene is nested in the intron of the PCG), the distance between the two genes, or also the gene id and the gene name of the closest gene (here the PCG) of the gene of interest are provided. For more details, see the column descriptions available on GEGA (hyperlink associated with ‘show/hide columns’).

For each lncRNA:PCG, lncRNA:lncRNA and PCG:PCG pair for which tissue expression was available, the Kendall correlation (τ) between the expression values across tissues was computed, providing a co-expression indicator between the two genes of the pair.

### Orthology and GO terms

Gene orthology between chicken, mouse and human as well as GO terms and phenotype description were extracted using BioMart (16) from Ensembl (V107). GO terms come from the ‘Gene Ontology’ database (17,18), one of the most complete and widely used ‘functional’ databases. Phenotype description refers to the OMIA database (19). The Ensembl reference database chosen for extracting this information allows us to maintain it easily via the ‘biomart’ API tool at each update.

### GTEx data analysis

A list of 23 equivalent tissues between human and chicken has been established, and the abbreviations and colour codes used are available on GEGA. The median gene level TPM for these 23 tissues from RNA-seq data of human GTEx Analysis V8 was used (available at https://gtexportal.org/home/downloads/adult-gtex/bulk_tissue_expression).

### Summary of the available information

For each gene, a total of 170 pieces of information is available and described in the column's description available on GEGA (hyperlink associated with ‘show/hide columns’).

## Results

### General description of the GEGA tool

**Figure 1.**
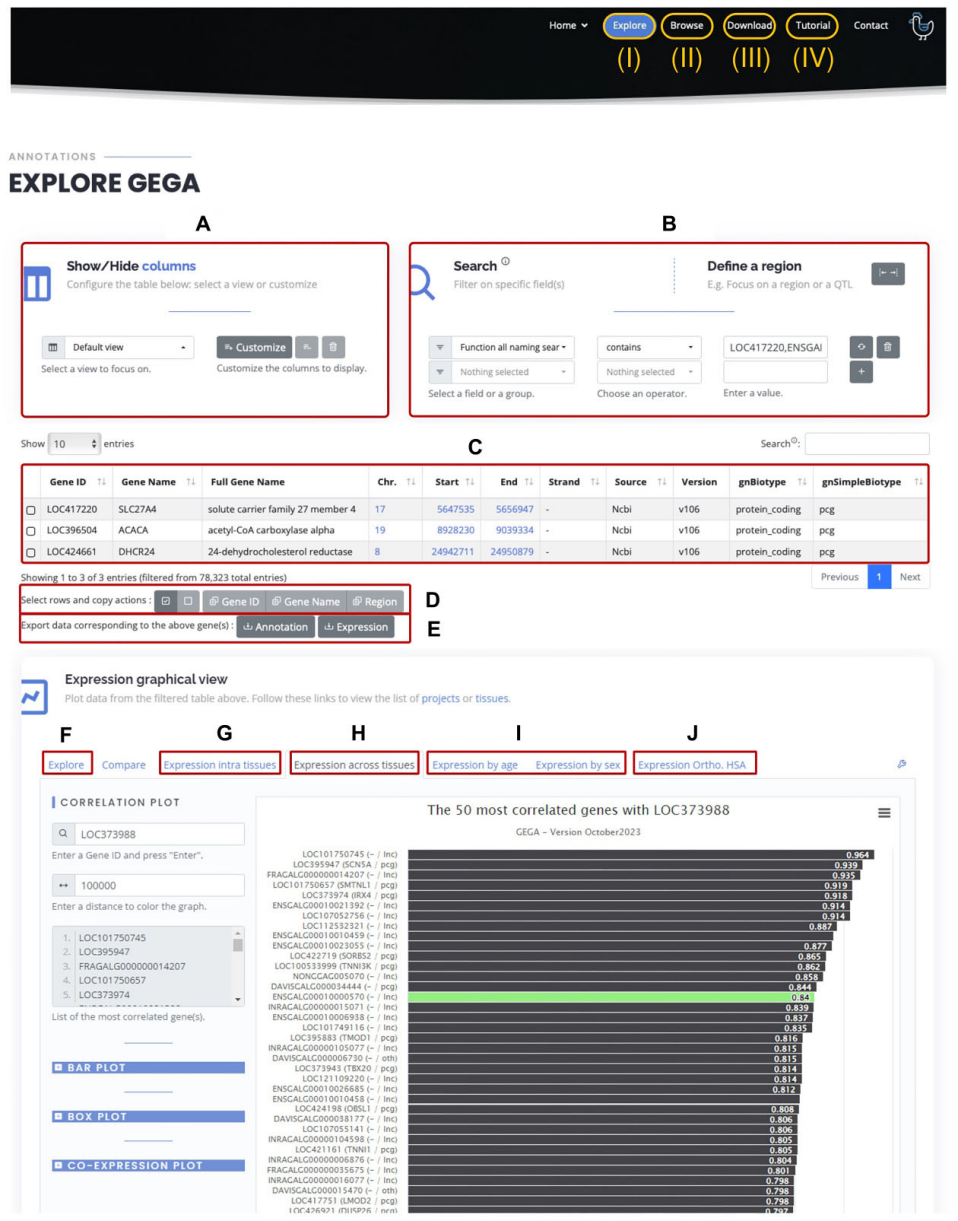
Interface overview. (I) GEGA main page to select genes of interest and observe their expression, including: (**A**) view selection panel; (**B**) filter selection panel; (**C**) table of the selected gene according to the chosen view; (**D**) copy panel; (**E**) exportation of annotation and/or expression; (**F**) visual exploration of the selected genes; graphical tab for (**G**) intra-tissue, (**H**) inter-tissues, (**I**) age/sex and (**J**) human orthologue expression. (II) Access to the browser to view the enriched atlas and the six annotations used to build it. (III) Download access to all files used in GEGA. (IV) Access to the tool presentation tutorials.

GEGA is an online tool designed to facilitate the exploration of an atlas of 78 323 gene models including both genomics and functional annotations based on the chicken GRCg7b genome assembly. Three main features are available (Figure 1): (i) an interactive table, in the upper part of the ‘Explore’ menu, allowing users to view various gene features, as briefly described in the Materials and methods; (ii) an interactive viewer, in the lower part of the ‘Explore’ menu, providing plots to explore the different patterns of expression and co-expression for gene models under various conditions (inter-tissues/intra-tissue; sex/age) but also to explore gene model information previously selected; and (iii) an interactive viewer in the ‘Browse’ menu to visualize gene models according to the six resources used for generating the gene enriched atlas. GEGA is publicly available at https://gega.sigenae.org/ and is free to use. It does not depend on cookie files or any credentials. The web server has been tested on Chrome V.116.0, Firefox V.116.0 and Safari V16.5.2. browsers. A dedicated contact tab is available for users to provide feedback, suggest improvements or report issues such as conception problems or bugs.

### Interactive table—data viewing

A total of 170 information columns are available in GEGA (see the ‘show/hide column link’ in the interface), but users may not need all of them. Therefore, by default, only data concerning the gene model *sensustricto* are initially displayed. Users can display additional data through the dedicated ‘Views’ panel (Figure 1A). Pre-filtered views have been implemented to guide new users and facilitate access to specific information following the main categories of information available in GEGA. The first three columns, which remain fixed, include the gene identifier from the source and the short and long gene names. The pre-configured views are as follows: (i) ‘Default’, displaying the origin, positional information and biotype; (ii) ‘All naming’, showing available identifiers for a gene model, particularly Ensembl and NCBI, as well as identifiers from previous assemblies such as galgal5 and GRCg6a; (iii) ‘All functional’, listing GO and phenotype terms for each gene and their associated orthologues; (iv) ‘Expression & Tissue-specificity’, indicating expression values (in TPM) in each of 47 tissues, highlighting top tissues and tissue specificity; (v) ‘Orthology’, showing orthologous relationships and equivalents with human and mouse for each PCG; (vi) ‘Gene structure’, detailing transcript, exon and intron structure; (vii) ‘FEELnc’ indicating the nearest gene model (PCG/lncRNA/miRNA) and configuration at the gene and transcript level, along with co-expression data at the gene level; and (viii) ‘Repeatability’, providing reproducibility information on loci representation across resources. For greater flexibility and specific usage, columns can be manually selected with the ‘Customize’ tool via their names or a tree structure with checkboxes.

### Interactive table—data filtering

Filters can be easily applied to extract specific sets of genes using the ‘Search’ panel (Figure 1B). All columns available in the ‘Views’ panel can be filtered and combined. Some filters can be applied simultaneously using the pre-defined ‘custom function’ available in the same selection menu. To date, the ‘Function all naming search’ and ‘Function all functional annot.search’ allow filtering across all naming (names and gene identifiers from both Refseq and Ensembl) or GO-related columns, respectively. Logical operators can be applied according to the data type: the ‘equals/not equals’ operator can be used for both numerical and categorical data, while ‘greater/lower’ is specific to numerical data and ‘contained/not contained’ to categorical data. For a given filter, the comma (‘,’) delimiter can be used to indicate multiple possibilities (OR operator). For example, applying the filter ‘gnSimpleBiotype – contains – lnc,pcg’ will result in the selection of both lncRNA and PCG. Similarly, ‘Function all naming search – contains – LOC417220,ENSGALG00010025549,ACACA’ will result in the selection of the three specified genes, even if the type of naming used varies. Each line corresponds to a criterion, and a set of criteria (AND operator) is considered as an intersection of conditions. Even if expression in each tissue is not available individually through the interactive table, filtering on tissue gene expression is possible using the ‘[tissueName] median’ filter. Note that filters defined in the ‘Search’ panel apply even if the column is not selected in the ‘Show/Hide columns’ menu.

To address specific usage need (see user cases), a ‘Define a region’ module has been designed to create a region surrounding a gene (using identifier or name) or position (defined by chromosome and position). This can be used, for example, for QTL analysis. Note that offsets can be applied equally or differently on each side.

### Interactive table—output data

After filtering, genes of interest (i.e. lines of the table, Figure 1C) can be easily selected, and gene names, gene identifiers or the genomic region can be copied to the clipboard (Figure 1D). The selected list of genes or the genomic region can then be pasted elsewhere for further analysis, in particular for interactive visualization. Two types of filtered tables are available for download (Figure 1E) as a *.csv* file, depending on user preference: (i) ‘Annotation’, which includes all available information or only what is displayed through the custom view as chosen by the user; and (ii) ‘Expression’ which provides tissue expression levels for the filtered genes in the 47 tissues.

### Interactive visualization—explore genes according to the filtered interactive table

An interactive visualizer provides a graphical overview of gene sets selected by specific filters (Figure 1F). This allows the generation of pie charts for categorical variables and boxplots for numerical variables. For two numerical variables, a scatter plot can be created to show potential relationships between them, e.g. lncRNA and PCG co-expression versus separating distance.

### Interactive visualization—global usage to explore gene expression

Expression patterns can be analysed either across 47 tissues or within a specific tissue, allowing observation of variation between individuals and project. Gene expression can also be examined according to factors such as sex [projects ‘FrAgENCODE’: *n* = 2 (9) and ‘RIRinrae’: *n* = 4] or age [projects ‘Kaessmann’: *n* = 4 (20) and ‘RIRinrae’: *n* = 4], where multiple tissues have been studied. Although each feature has its own specificity, boxplots and co-expression plots (scatter plots) are always available and function similarly. For boxplots, users can input a gene or list of genes (separated by spaces or commas), and expression for each gene will be displayed in the order of input. To enable comparison, expression can be unified by displaying all values on a scale from 0 to the maximum observed across all genes. Sample identities (individuals) can also be displayed. Tissues are classified by abbreviation, by default, but can be ordered by expression proximity as analysed in Degalez *et al.* (3). Tissues sharing colour ranges generally share a physiological system. For co-expression plots, a reference gene identifier is provided first, followed by target gene(s). Each plot of the reference (*x*-axis) versus target (*y*-axis) gene is displayed initially in input order. However, plots can be ordered by correlation (from 1 to –1) or filtered by a correlation threshold. Since co-expression can follow different patterns, both linear and logarithmic plots are available, and displaying the regression line is also an option.

### Interactive visualization—specific usage: ‘expression intra-tissues’ (Figure 1G)

First, after selecting the tissue of interest, expression profiles across 47 tissues can be displayed for the 40 most highly expressed genes, helping to identify tissue-specific or highly expressed ubiquitous genes. Second, boxplots of expression for each project related to the chosen tissue, or co-expression plots between gene pairs of interest across all samples from all projects related to the chosen tissue can be displayed.

### Interactive visualization—specific usage: ‘expression across tissues’ (Figure 1H)

First, to overview gene expression correlation across 47 tissues, a correlation plot is available for one gene of interest. Once the gene identifier is provided, the 50 most correlated genes are displayed in descending order of correlation. The gene list can be accessed and copied/pasted for further investigation. Note that genes on the same chromosome as the gene of interest appear in blue, and those within a specified distance threshold appear in green. This helps identify clustered co-expressed genes, regardless or not of the availability of chromosomic location. As an example, TBX5 (LOC373988; chr15:12 317 331) shows a high co-expression across tissues (ρ = 0.84) with a lncRNA (ENSGALG00010000570; chr15:12 314 252) which is in 15th position and coloured in green because the genes are separated by <100 kb (default value).

For examining expression across tissues, after providing a gene or a gene list, either a boxplot (as described before) or a barplot of expression across the 47 tissues can be generated. Boxplots consider all samples from all projects together, plotting all available data. Barplots first calculate the median expression for each project and then the median of those medians, thus lacking sample resolution. Barplots have the same options as boxplots.

### Interactive visualization—specific usage: ‘expression by age/sex’ (Figure 1I)

First, a project of interest must be selected from those related to multiple ages (projects ‘Kaessmann’ and ‘RIRinrae’) or sexes (projects ‘FrAgENCODE’ and ‘RIRinrae’). This ensures reliable and consistent subgroup comparisons across studied factors. Next, the tissue of interest has to be selected among the tissues available for the selected project. Finally, barplots of gene expression across samples can be generated according to the factor levels (ages or sexes) on linear or logarithmic scales, with unified or raw maximum values. Boxplots can be generated for a specific tissue as barplots, but also for different tissues, up to four tissues. Boxplots can be ordered by condition or tissue, depending on what the user wants to highlight. Finally, co-expression plots using all available samples for a given tissue of a selected project can be displayed, following the previously described process.

### Interactive visualization—specific usage: expression ortho.HSA (Figure 1J)

For chicken genes with a human orthologue, expression in both species can be observed across 22 equivalent tissues. After inputting the chicken gene identifiers, the associated one-to-one human orthologues are retrieved, and expression of both can be displayed either (i) through a stacked barplot for a side-by-side comparison, with the option to group tissues according to their functional proximity, as defined previously, or (ii) through a co-expression plot to observe overall expression patterns. For a given gene, the raw expression values for both species can be download as a *.csv* or *.xls* file.

### Interactive visualization—output data

Regardless of the plot type, the associated figure can be downloaded in the formats PNG, JPEG, SVG or PDF through the dedicated menu (top-right of plot). The data used for plotting can be downloaded as a *.csv*, formatted to enable re-plotting with specific tools (e.g. R-base or ggplot). Currently, the production of multiplots is unavailable; each plot and its associated data must be downloaded independently.

### Interactive browser

The ‘Browse’ menu displays a genomic region or gene of interest along with surrounding genes at the chosen resolution. Users can observe gene models present in the enriched atlas, as well as those from each of the six resources used to generate it, indicated by a specific colour. Gene models are presented with their respective orientations similar to a classical genome browser, allowing visualization of FEELnc gene pair configurations as indicated in the interactive table. For example, TBX5 (LOC373988; chr15:12 317 331) can be observed in a divergent orientation with its lncRNA partner (ENSGALG00010000570; chr15:12 314 252).

### GEGA usages through typical user cases

**Figure 2.**
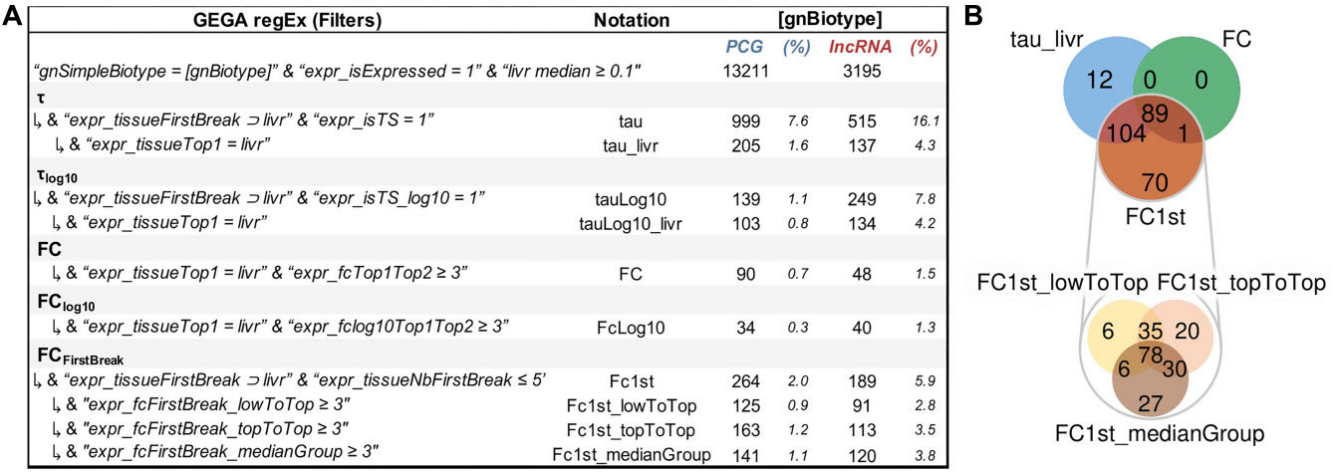
Tissue-specific (TS) metrics. (**A**) Number of PCGs (blue) and lncRNAs (red) considered as TS in the liver according to the various metrics available in GEGA. (**B**) Intersection of the TS metrics for PCGs in the liver.

- ***Focus on tissue-specific (TS) analysis* (*Figure 2***)

For genes of interest, tissue specificity can provide additional information depending on the dataset used and the metrics applied within GEGA. Different metrics are available. (i) The tau metrics, τ: ‘expr_tau’ with raw expressios or τ_log10_: ‘expr_tauLog10’ with expression in log10, and ‘expr_isTS’ and ‘expr_isTS_log10’ indicating a τ and τ_log10_ ≥ 0.90, respectively. These tau metrics provide a unique score relative to the expression pattern across the 47 tissues. (ii) The FC between the two most expressed tissues (‘expr_fcTop1Top2’). Note that a τ ≥ 0.90 corresponds to an average and median FC of 71 and 2.5, respectively, for tissues with expression ≥ 1 TPM in at least one tissue, and 5 and 2.19 for tissue with expression ∈ [0.1,1[ TPM. However, as already mentioned, the τ and FC metrics correspond to a unique tissue, but a gene can be TS for several tissues which are functionally close. Therefore, the concept of ‘break’ was introduced, allowing distinguishable tissue groups to be separated according to the expression pattern. The list of tissues and the total number included in the first group can be observed using the ‘expr_tissueFirstBreak’ and ‘ expr_tissueNbFirstBreak ’ filters, respectively. (iii) Consequently, three metrics have been defined to quantify the expression FC between both groups delimited by the first break (‘expr_fcFirstBreak_lowToTop’, ‘expr_fcFirstBreak_topToTop’ and ‘expr_fcFirstBreak_medianGroup’; see the Materials and methods for details). As an example, results of each metric on both expressed PCGs and lncRNAs in the liver are available in Figure 2. Consistent with previous findings in chickens (3,11) and other species (21), lncRNA genes exhibit a higher tissue specificity compared with PCGs. Each TS metric exhibits unique characteristics, with varying numbers of TS genes shared across methods. As an example, the 90 TS genes detected by the ‘FC’ metric are included in the ‘tau_livr’ metric (*n* = 205), which itself is 94% included in the ‘FC1st’ metric (*n* = 264). The latter identifies three distinct TS groups based on FC calculation methods (*n* = 125, *n* = 163 and *n* = 141). Intersection results among TS metrics can be visualized via Venn diagrams produced in the ‘Compare’ tab of GEGA’s graphical view or in Supplementary Figure S1 for this example.

**Figure 3.**
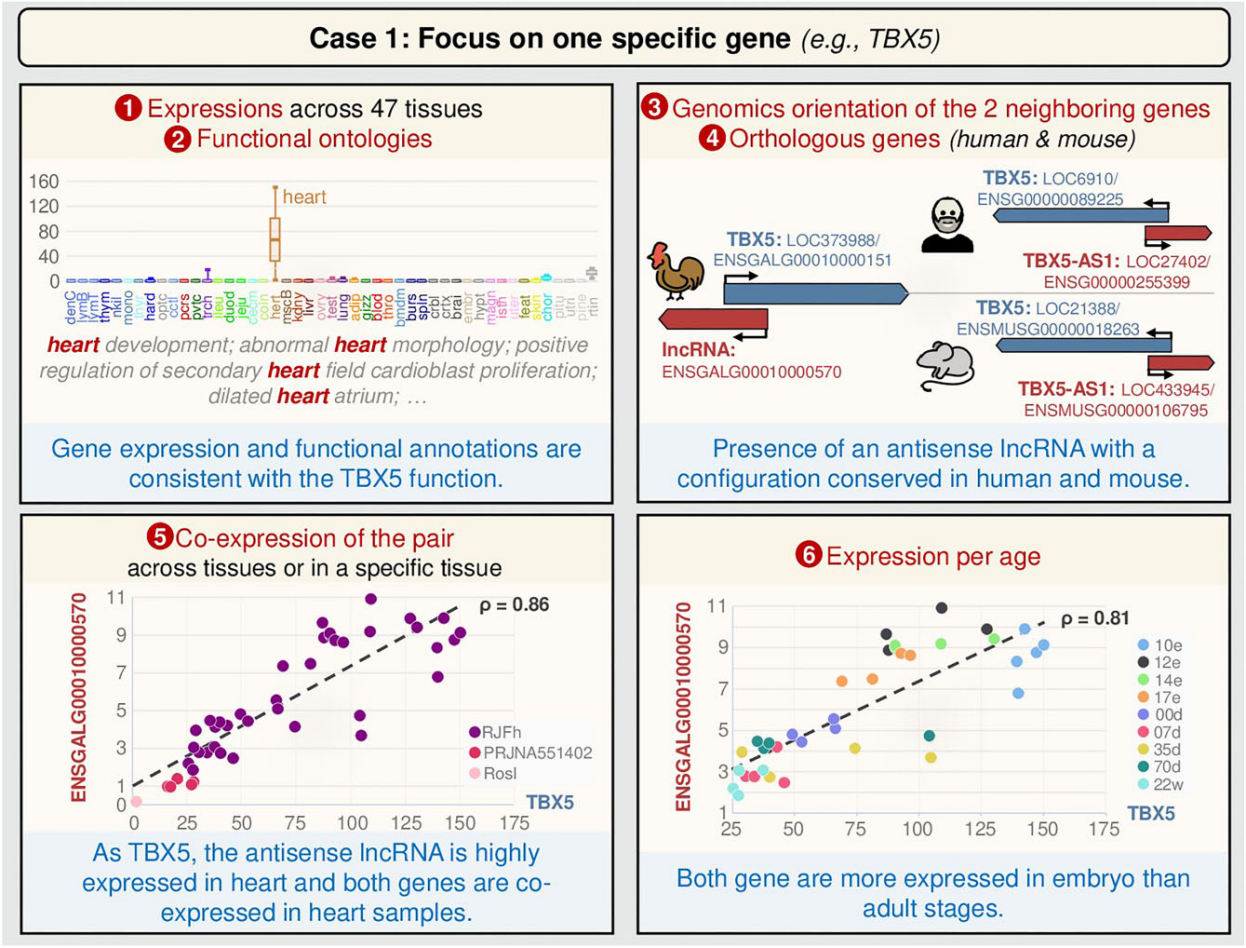
User case 1: GEGA for exploring a specific gene, its genomic environment and its potential functions (e.g. TBX5). Correspondence between full tissue names and the four-letter abbreviations is available on GEGA (hyperlink associated with ‘tissues’ in the ‘Expression Graphical View’). Additional project-specific information is available on GEGA via the hyperlink associated with ‘projects’ in the ‘Expression Graphical View’. e, embryonic day; d, day; w, week.

- ***Focus on one specific gene (e.g. TBX5; Figure 3)***

TBX5, a T-box transcription factor (22), plays a crucial role in vertebrate cardiac morphogenesis. Studies have demonstrated that overexpression of TBX5 in embryonic chick hearts inhibits myocardial growth and cardiogenesis, thereby participating in modulating vertebrate cardiac growth and development (23). Using GEGA, this gene can be explored comprehensively, e.g. following this process: (i) analysis of expression across the 47 tissues and (ii) through functional terms (GO terms/phenotype terms) to underline those consistent with the TBX5 function. (iii) Examination of genomic configuration between the two neighbouring genes, highlighting the presence of an antisense lncRNA. (iv) Gene orthology analysis with human and mouse revealing an orthologous PCG. This initial observation served as manual verification of the conservation in both species of the antisense lncRNA. (v) Analyses of expression and co-expression across the 47 tissues showing that TBX5 and its antisense lncRNA are highly expressed in the heart and exhibit significant co-expression. (vi) Analyses of expression patterns across different ages, indicating expression of the gene pair in embryos compared with adults.

This data exploration using GEGA suggests that the antisense lncRNA (known as TBX-AS1 in human) could be a regulator of TBX5 or at least could share a common function. This result aligns with the fact that the cardiac transcription factor gene (TBX5) is associated with a bidirectional lncRNA, as reported in 2018 by Hori *et al.* (24).

**Figure 4.**
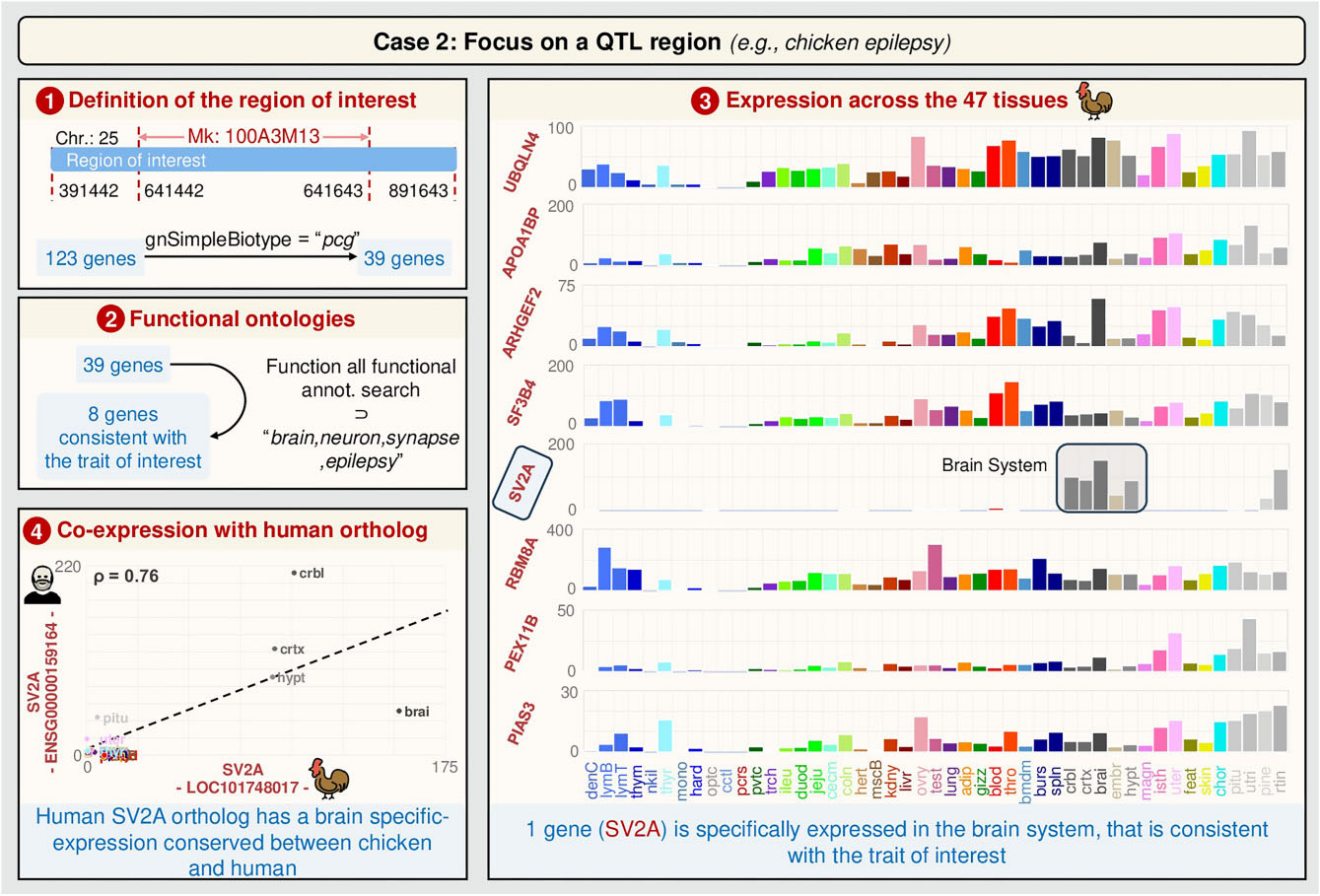
User case 2: GEGA to analyze a QTL region (e.g. epilepsy region around the marker 100A3M13). Correspondence between full tissue names and the four-letter abbreviations is available on GEGA (hyperlink associated with ‘tissues’ in the ‘Expression Graphical View’). Chr., chromosome.

- ***Focus on a QTL region (e.g*.*chicken epilepsy; Figure 4***)

In 2011, a QTL for epilepsy trait in chicken was mapped around the markers 100A3M13 and SEQ1009, that mapped at that time to linkage group E26C13 which has been identified thereafter as the micro-chromosome GGA25 (25). Through fine mapping and other molecular approaches, the authors identified the SV2A gene as associated with the epilepsy trait. GEGA could have facilitated the identification of this gene as follows. (i) Definition of a region of ± 250 kb around 100A3M13 (i.e. chr25 from 641 442 to 641 643) and display of all the genes, with the possibility of filtering the biotype of the genes (PCG, lncRNA, etc.). In the current atlas, 39 PCGs are observed in this region. (ii) Analyses of the functional terms associated with these 39 genes. Eight of these genes are associated with GO terms or phenotypes related to the trait of interest, i.e. ‘brain, neuron, synapse, epilepsy’. (iii) Examination of the expression patterns across the 47 tissues. Only SV2A shows specific expression in the cerebral system, consistent with the trait of interest. (iv) Observation of the orthologous gene expression in human, revealing that the expression pattern is conserved between both species.

**Figure 5.**
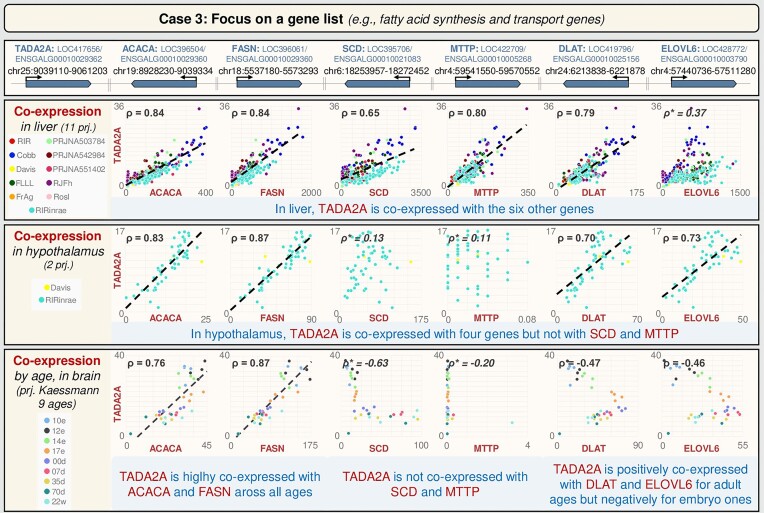
User case 3: GEGA for exploring a gene list (e.g. fatty acid synthesis and transport genes). The regression line is not shown and the correlation value is indicated in italics and with a (*) if the correlation is ≤ |0.2| or if a group effect is observed. Additional information on projects is available on GEGA via the hyperlink associated with ‘projects’ in the ‘Expression Graphical View’. e, embryonic day; d, day; w, week. chr., chromosome; prj., project.

- ***Focus on a gene list (e.g. fatty acid synthesis and transport genes; Figure 5)***

The main role of the liver in the chicken adaptive response to a switch in dietary energy source through the transcriptional regulation of lipogenesis was previously reported. Previous studies have identified 298 down-regulated genes in a ‘high fat/high fibre diet (HF)’ compared with a standard ‘low fat/high starch diet’ (LF), with an enrichment of genes related to fatty acid synthesis and transport (26). Among these genes, TADA2A (Transcriptional Adaptor 2A) showed highly correlated expression with key enzymes of fatty acid (FA) anabolism such as ACACA, FASN, SCD, DLAT, MTTP and ELOVL6. Consequently, TADA2A has been identified as a potential new player in the regulation of lipogenesis. Further investigation of TADA2A’s co-expression with these six genes of interest was conducted using GEGA across 11 liver-related projects comprising 265 samples. The analysis confirmed significant hepatic co-expression (0.65 ≤ ρ ≤ 0.87) of TADA2A (LOC417656/ENSGALG00010029362) with ACACA, FASN, SCD, MTTP, DLAT and ELOVL6 (LOC396504/ENSGALG00010029360, LOC396061/ENSGALG00010029360, LOC395706/ENSGALG00010021083, LOC422709/ENSGALG00010005268, LOC419796/ENSGALG00010025156 and LOC428772/ENSGALG00010003790, respectively), with particularly high correlations observed for ACACA and FASN (ρ ≥ 0.83). The co-expression in brain was then explored, given the essential role of FAs in brain functions (27) and due to a co-expression between TADA2A and ACACA in mouse brain already reported (26). In the hypothalamus (two projects), TADA2A is highly co-expressed with ACACA, FASN, DLAT and ELOVL6, but not with SCD and MTTP (ρ ≤ 0.15). Analysis of age-related co-expression patterns in the brain, using the ‘Kaessmann’ project, revealed that TADA2A is highly co-expressed with ACACA and FASN across both embryo and adult ages. Specifically, TADA2A showed positive co-expression across adult ages and negative co-expression across embryo ages with DLAT and ELOVL6, while it did not exhibit significant co-expression with SCD and MTTP. In summary, GEGA analysis demonstrates that TADA2A shows high co-expression with ACACA and FASN, genes encoding the two first key enzymes of lipogenesis, whatever the projects, the ages and the analysed tissues (liver or brain/hypothalamus), whereas the co-expression pattern varies across these different conditions/tissues with DLAT, ELOVL6 and SCD.

## Discussion

The GEGA tool presented in this article integrates and synthesizes the gene models from the reference NCBI-RefSeq and Ensembl/GENCODE databases, along with those from international collaborative projects such as FAANG and NONCODE (3). Usually, the scientific community interested in chicken genes handle either NCBI-RefSeq or Ensembl/GENCODE identifiers, i.e. LOCxx or ENSGALGxx identifiers, respectively. One strength of GEGA is that it accommodates both identifiers and also the HGNC gene name. However, for genes existing in both reference databases, user-input ENSGALGxx identifiers may not correspond to a gene model due to default matching to an NCBI RefSeq identifier. Users must use the ‘Function all naming gene’ function (that gathers all the gene naming columns) to correctly identify the corresponding gene model. The integration of the six annotation databases not only expands the number of gene models but also enables assessment of their consistency across databases. Note that it can be difficult to assess the accuracy of the gene models, particularly in terms of exon/intron structure or gene fusions/splits due to different factors such as short-sequencing RNA-seq samples, the variety of bioinformatics analysis methods used for the gene modelling or also the impossibility to gather all the condition-specific RNA-seq samples with a high enough sequencing depth. Nonetheless, the presence of a gene model across multiple databases supports its existence, even if the isoforms supporting these models are still badly described in farm species (2,3,28). Moreover, the observation of expression in at least one of the 47 tissues is another argument for the existence of a gene model, especially for lncRNAs, which are weakly expressed and sometimes supported by only a few reads. Indeed, a second strength of GEGA is to provide the expression profiles of these genes across 47 tissues using 1 400 samples grouped into 36 datasets that encompass the diverse physiological systems of chickens, including some bird-specific tissues, e.g. the different segments of the oviduct that produce the egg white and shell. As a result, 63 513 out of the 78 323 genes (i.e. 81%) exhibit expression (normalized TPM expression ≥ 0.1) in at least one of these 47 tissues. Note that some neighbouring genes in the same strand orientation may correspond to the same gene, as already demonstrated in Muret *et al.* (29). The observation of significant co-expression across these 47 tissues and within the various tissues may indicate such cases, thus inviting caution in biological interpretations. This standardized functional annotation greatly facilitates transcriptomic studies in this species by providing a reliable expression atlas reference. This resource is complementary to the chicken EBI-Expression Atlas (30) used by Ensembl, which covers 26 tissues (including five embryonic tissues). However, among the 39 projects, which all referred to previous genome assemblies, only five provide easy access to expression data (baseline project), while the remaining 34 projects involve differential expression experiments where only FCs between conditions are available. Note also that 20 projects used Affymetrix technology, which does not include new gene models. Furthermore, GEGA’s interactive graphical interface facilitates exploration of expression datasets across different tissues combined with physiological conditions such as sex and development stages, enabling analysis of various tissue specificity indicators, paving the way for new discoveries. The availability of such indicators, ranging from basic metrics such as FC to more sophisticated measures such as tau metrics, allows GEGA to address inquiries from biologists seeking to contextualize gene expression across all chicken tissues, considering the broad tissue panel in this study. The possibility to explore co-expression between different genes of interest is also an added value. However, gene expression data in GEGA do have limitations, such as occasionally incomplete metadata, which restricts certain analyses, e.g. for developmental stages or sex, that are provided inconsistently across tissues. This limitation may be overcome in the future by adding new projects (suggestions can be made through the ‘Contact’ tab): new well-defined metadata could easily be added in a coherent and standardized manner using the nfcore ‘rnaseq’ pipeline (31) in conjunction with the *.gtf* file associated with this gene-enriched atlas (3). Furthermore, the incorporation of GO terms and orthologous relationships with mice and humans combined with co-expression between human and chicken species provides valuable functional insights into the interpretation of genomic data. The chicken serves as a model species for studying numerous human diseases, and comparisons of expression profiles between species provide a wealth of information. Similarly, exploring co-expression networks between coding and non-coding genes highlights potential transcriptional regulation and opens up promising research prospects. However, current reference databases only provide orthologous relationships between PCGs and a limited number of miRNAs, information that has been integrated in GEGA. The integration of lncRNA orthology into GEGA will progress alongside advancements in this field. One of the issues with GEGA is maintaining it up to date as genome assemblies evolve, in particular with the emergence of complete telomere-to-telomere genome sequences (T2T) (32). The choice was made to update the GEGA annotation only when new versions of genome assemblies are used as reference in the NCBI-Refseq and Ensembl/ENCODE databases, while facilitating the transition from one assembly to another and allowing users to work with older versions. For a given assembly (currently GRCg7b), versions of the reference gene model annotations are fixed (V106 for NCBI-RefSeq, the latest available, and V107 for Ensembl/GENCODE, equivalent to the actual version v110). In conclusion, despite these limitations, GEGA already provides a robust analytical framework for functional exploration of the chicken genome. Coupled with advancements in genome assembly and annotation of gene models, this bioinformatics tool supports the functional characterization of the transcriptome of this model species and paves the way for enhanced understanding of complex genomes.

## Supplementary Material

lqae101_Supplemental_File

## Data Availability

The data underlying this article are available via the GEGA website at https://gega.sigenae.org/ and through the ‘Download’ tab. The source code used for the server-side implementation is available on GitLab at https://forgemia.inra.fr/philippe.bardou/gega.
