# Supplementary material for: GEGA (Gallus Enriched Gene Annotation): an online tool providing genomics and functional information across 47 tissues for a chicken gene-enriched atlas gathering Ensembl and Refseq genome annotations
Source: NAR Genom Bioinform. 2024 Aug 16;6(3):lqae101. doi: 10.1093/nargab/lqae101 (PMC11327871; doi:10.1093/nargab/lqae101)
Supplement: lqae101_Supplemental_File [file lqae101_supplemental_file.pdf]

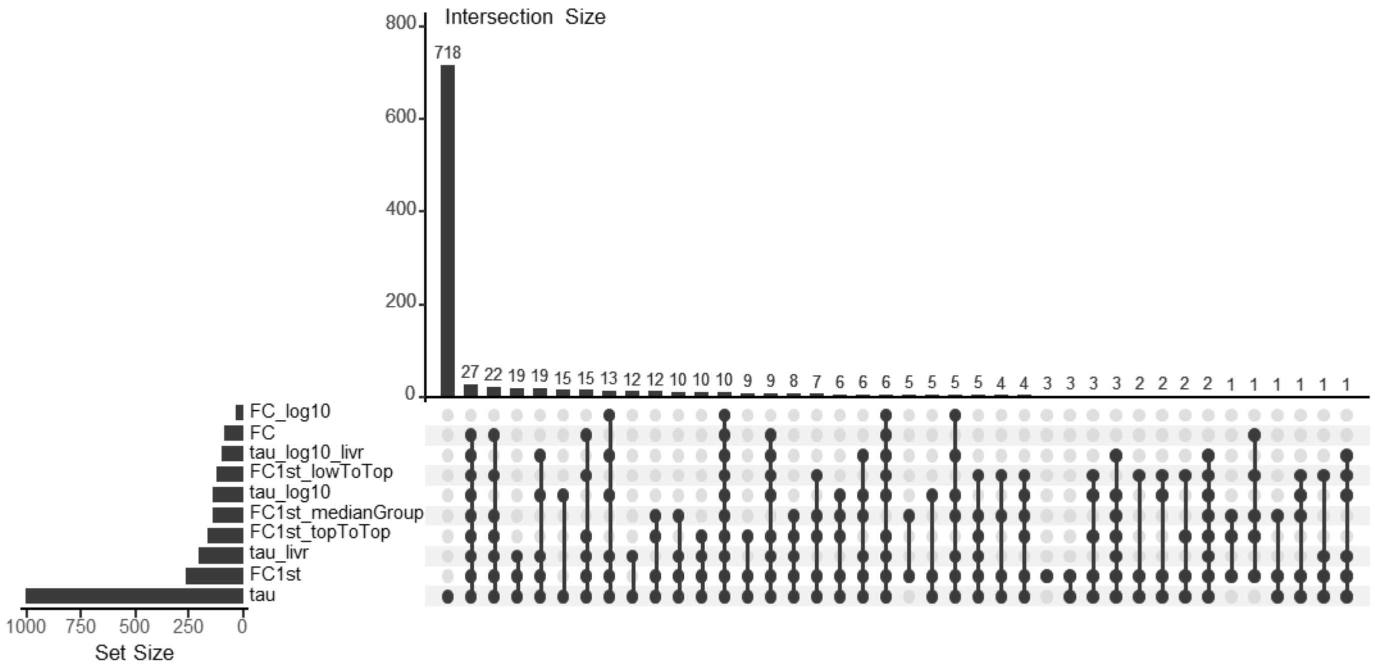


**Supplementary Figure 1. Intersection of the number of tissue-specific PCGs in the liver detected by the different tissue-specific metrics available in GEGA**. The notation used for tissue-specific metrics are available in Figure 2.
